# Soil Moisture Sensor Information Enhanced by Statistical Methods in a Reclaimed Water Irrigation Framework

**DOI:** 10.3390/s22208062

**Published:** 2022-10-21

**Authors:** Anthony Giorgio, Nicoletta Del Buono, Marco Berardi, Michele Vurro, Gaetano Alessandro Vivaldi

**Affiliations:** 1 Ernst & Young, 70126 Bari, Italy; 2 Dipartimento di Matematica, Università degli Studi di Bari Aldo Moro, Via E. Orabona 4, 70125 Bari, Italy; 3 Istituto di Ricerca sulle Acque, Consiglio Nazionale delle Ricerche, Via F. De Blasio 5, 70132 Bari, Italy; 4 Dipartimento di Scienze Agro Ambientali e Territoriali, Università degli Studi di Bari Aldo Moro, Via G. Amendola 165/A, 70126 Bari, Italy

**Keywords:** soil water content, soil salinity, time series, irrigation with reused water, infiltration

## Abstract

Time series modeling and forecasting play important roles in many practical fields. A good understanding of soil water content and salinity variability and the proper prediction of variations in these variables in response to changes in climate conditions are essential to properly plan water resources and appropriately manage irrigation and fertilization tasks. This paper provides a 48-h forecast of soil water content and salinity in the peculiar context of irrigation with reclaimed water in semi-arid environments. The forecasting was performed based on (i) soil water content and salinity data from 50 cm beneath the soil surface with a time resolution of 15 min, (ii) hourly atmospheric data and (iii) daily irrigation amounts. Exploratory data analysis and data pre-processing phases were performed and then statistical models were constructed for time series forecasting based on the set of available data. The obtained prediction models showed good forecasting accuracy and good interpretability of the results.

## 1. Introduction

Agricultural and environmental sciences are increasingly making use of data-driven methods to accomplish their goals and improve their assessments. Here, we focus on the problem of irrigation with reclaimed water on farms in semi-arid regions (see [1,2]). This issue has an obvious agronomic relevance but it is also of significant interest from the environmental point of view in a circular economy framework, in which natural resources find new lives after their primary use.

In this context, using reclaimed wastewater for irrigation purposes allows to reduce water pumping from stressed groundwater aquifers, which are the primary sources of water in semi-arid regions, such as the Apulia region (for instance, see [3]).

The accurate description and forecasting of soil features (water content, salinity, temperature) are crucial for correctly managing irrigation and fertilization and can be accomplished on a larger scale using more highly engineered methods (e.g., [4]). Recent papers have focused on models for optimizing water consumption during irrigation (for instance, see [5]) or controlling irrigation (as in [6]). To this purpose, it is worth recalling that the prediction of water content dynamics in soils is generally accomplished using physical models based on Darcy’s law and mass balance arguments, which rely on the Richards equation. Since these models seldom produce analytical solutions (for instance, see [7,8,9]), numerical methods and solutions are generally still proposed in the literature, sometimes highlighting features in heterogeneous soils [10,11,12,13], sometimes the proper treatment of nonlinear issues (e.g., [14,15,16]) and sometimes elegant techniques for dealing with time or space discretization (e.g., [17,18]). More refined models and software are able to couple moisture content dynamics with the heat equation (as in [19]). On the other hand, the use of physics-based models requires a deep the knowledge of soil characteristics and significant laboratory efforts for assessing hydraulic parameters (for example, see [20,21]), the measurement of which can be expensive and time-consuming. Thus, due to both the practical difficulties in accurately and reliably measuring soil hydrological properties in laboratory experiments and the numerical difficulties in solving the Richards equation, in this paper, we consider a class of data-driven models, i.e., we focus on forecasting soil water content (SWC), soil salinity and soil temperature with a time horizon of 48 h, which could offer sufficient advances for properly scheduling irrigation.

Starting from the available time series data with different time resolutions, a pre-processing phase was performed to increase data quality and overcome the presence of some missing values. The improved data were used to obtain forecasts by implementing different statistical methods, such as autoregressive integrated moving average models and their extensions, to explicitly support the presence of seasonal components in the time series data. Discussions about the potentialities and drawbacks of the proposed statistical forecasting mechanisms are also highlighted in this paper. Although irrigation with reclaimed water is fast becoming an increasingly compelling issue (for instance, see [22,23,24,25,26]), in the context of irrigation with reused water from agro-industrial origins, the use of this type of statistical technique is novel. Precisely, this paper illustrates how these statistical techniques constitute user-friendly data-driven approaches that can be used simply to obtain forecasts using data from standard soil probes.

## 2. Materials and Methods

### 2.1. Study Site and Data Source

The test site was located in an agricultural area in the Foggia district (Stornarella: 41°15′ N, 15°44′ E) of the Apulia region in southern Italy. The site belongs to the Fiordelisi agricultural and food manufacturing company, which produces and processes vegetables.

On this farm, an experimental area of $3000\;m^{2}$ was used for the cultivation of tomatoes plants, which was divided into nine different blocks with plants that were irrigated with two water sources: groundwater and treated agro-industrial wastewater. The site is mainly characterized by a Mediterranean climate, with a long-term mean annual rainfall of 590 mm, which is mainly distributed from October to April. The time series data used in this experiment were from three different sources:

1. soil data, i.e., soil water content (%), soil temperature (°C) and soil salinity (mS/cm) at depths of 30 and 50cm that were measured continuously by two probes with a 15 min frequency;
2. irrigation data, i.e., the daily volume ($m^{3}$) of water employed to irrigate the tomatoes plants in each treatment block;
3. atmospheric data, i.e., the values of rainfall (mm), temperature (°C), humidity (%), radiation and evapotranspiration (mm) that were recorded by a weather station (Stornara) near to the experimental area.

**Figure 1:**
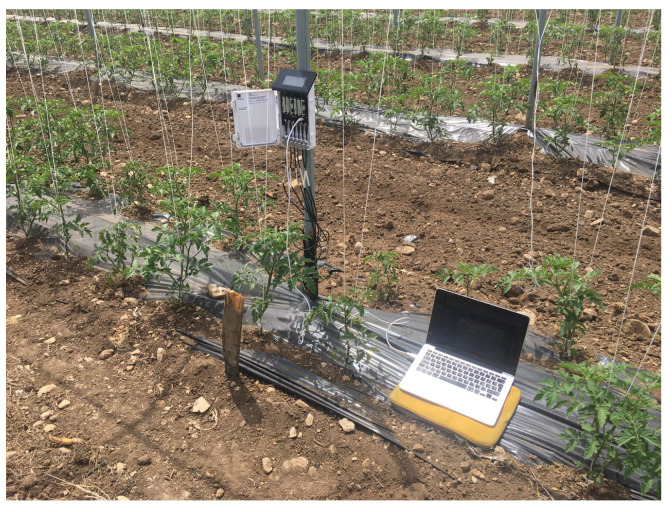
The control unit downloading TDR data from the Fiordelisi field site.

Data were collected during the 2019 irrigation season (see Figure 1), starting in June and ending in early September. In this paper, we only consider the data related to August 2019. In this experiment, the data were recorded using TEROS 12 sensors, provided by METER, which are able to measure soil moisture, temperature and electrical conductivity (EC). The data logger that was used was a ZL6Pro.

### 2.2. Datasets

The datasets used were provided by the Smart Water project, in which the CNR-IRSA and the University of Bari Aldo Moro were involved. The experimental study concerns the data collected in August 2019 during the irrigation season. Three different irrigation treatments were compared:

**A:**  fresh water (FW) + conventional fertigation;
**B:**  agro-industrial wastewater (AIW) + conventional fertigation;
**C:**  AIW + smart water fertigation.

**Figure 2:**
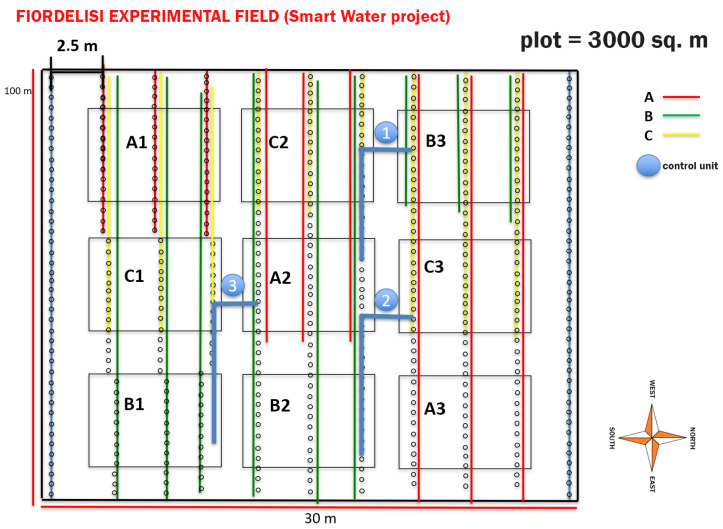
The subdivision of the agricultural area under study on the Fiordelisi farm, which cultivated tomatoes. The area was divided into nine production blocks, the irrigation treatment for each of which is indicated.

Figure 2 shows the arrangement of each block and its treatment. As can be observed, each treatment had three replicates. The available data offered various information on the soil, irrigation and atmospheric parameters of the site under study. The data were stored in three datasets, which were characterized by the following features:

- **soil data**, i.e., the soil water content (%), salinity (mS/cm) and temperature values (°C) at 30cm and 50cm depths in the A2, B3 and C2 blocks (Figure 2) that were collected continuously every 15 min (for a total of 73 out of 96 daily surveys) over the time period of 5 August 2019 11:15:00–18 December 2019 9:30:00;
- **irrigation data**, i.e., the water volume ($m^{3}$) used for irrigation in each treatment block that were recorded daily over the time period of 1 June 2019–31 August 2019 and organized in an Excel file;
- **climate data**, i.e., data collected by the Stornara weather station, located near the farm, over the time period of 1 January 2019 00:00:00–3 June 2020 23:00:00. These data referred to hourly surveys of the quantities of:
  - precipitation (mm);
  - temperature (°C);
  - humidity (%);
  - radiation (Mj/mq);
  - wind (speed, gust (m/s) and direction);
  - upper and lower leaf wetness;
  - atmospheric pressure (hPA);
  - dew point (°C);
  - Etp (mm).

Some additional considerations about the influence of the sensor choice on the final statistical model need to be addressed. Sensor sensitivity is a characteristic that discriminates one sensor from another. Sensor measurements can apparently only be performed under the same conditions as uncontrolled quantities to which sensors are even minimally sensitive may alter the measurement process. In this context, registered data depend on the accuracy of the sensors used to measure them, so data acquired from two different sensors may be characterized by different data quality and precision. Moreover, when a sensor is positioned outdoors or generally not in a completely controlled area, several problems can occur and undermine the quality of the collected data. Problems involving the completeness of the collected data, missing values, anomalies and outliers frequently occur during real observations [27]. The final forecasting results depend on the nature of the model and strongly on data quality. When high-quality software programs are used to implement forecasting methods based on the least squared regression problem, the well-conditioned properties of the data matrices generally induce the well-conditioned properties of the final numerical models.

### 2.3. Data Pre-Processing and Analysis

All data were collected as CSV files and uploaded into an R environment using a Jupiter Notebook with the Anaconda Suite. In this paper, we focus on the analysis and forecasting of water content and salinity data from the A2 block at a depth of 50 cm, but the same experimental results could be replicated for other blocks and other depths. The soil, irrigation and atmospheric data resulted in different data formats and lengths. A preliminary pre-processing phase of data integration and matching was performed to align all data values. This procedure is detailed in the following subsections.

**Figure 3:**
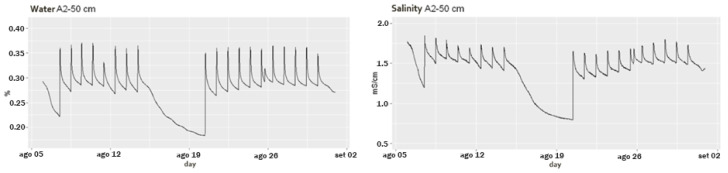
Soil water content and salinity in the A2 block at a depth of 50 cm, from 6 August 2019 00:00:00 to 31 August 2019 23:00:00.

To find an appropriate model to predict the data, we had to first understand whether time series data follow specific patterns in seasonality or trends. According to Figure 3, there was a decreasing trend when irrigation was not performed and a strong daily seasonal component due to irrigation was present for the time series data for both soil water content and salinity. Moreover, we noticed a double peak on 25 August, the second of which was related to a rainfall event. Consequently, each observation was highly correlated with the previous time series values.

### 2.4. Pre-Processing, Uploading and Visualization of Data in R

#### 2.4.1. Soil Data

The pre-processing and data visualization phases were carried out using the R libraries readxl and ggplot2. These phases highlighted the time period of 6 August 2019 00:00:00–30 September 2019 23:00:00, in which no missing values occurred, and compared the values from the various treatments.

By increasing the data resolution (extracting hourly values to adapt to the weather and irrigation data), a historical series with 600 observations was obtained. This showed a strong daily periodicity and sudden changes in the averages occurred when the plants were irrigated. We took this seasonality into account in the proposed models.

#### 2.4.2. Climate Data

The atmospheric data covered the same time period. The presence of some missing values required their reconstruction through the use of a function interpolating the values of the previous hour and those of the following hour. An interesting aspect concerned the presence of a peak in the volume of rain between 25 and 26 August. This information was integrated into the predictive analysis of soil water content and salinity at that time. A discrepancy of one hour occurred between the observed rain volume and the corresponding increment in the soil water content value, which could be explained by the time required for the rain to travel through the partially saturated soil. This complex infiltration phenomenon can be physically described by the Richards equation (for instance, see [28,29]). For the sake of simplicity, this discrepancy was corrected accordingly by shifting the values.

#### 2.4.3. Irrigation Data

Unlike the previous data, the irrigation data were recorded with a daily frequency. This required a readjustment to allow for a fair comparison to the other two hourly time series. For this purpose, a preliminary exploratory analysis of the SWC variable in the soil dataset was carried out by identifying, day by day, the times in which there was a significant increase in the SWC due to irrigation. This characteristic is reflected by the peaks in the left-hand panel of Figure 3. For most days, this peak occurred around 10:00–11:00 or 12:00–13:00. An hourly data frame was initialized to show the quantities of water ($m^{3}$) used for irrigation during the two hours following the occurrence of the peaks and a null value for all other times. In the case of the absence of the peaks, indicating a failure in the irrigation, the value was initialized with a null value. Null values (erroneously referred to as water content peaks) were replaced with the average value of the peaks from the previous and subsequent days.

### 2.5. Preparing Data in R

The forecast model for soil water content was implemented in the R environment, making use of the specific functions stats, astsa, tseries, forecast and zoo packages to manage the time series. The analyzed data were converted into class ts (time series) to be intrinsically defined by the seasonal frequency associated with that time series (in this case, daily and hourly), as well as their own beginning and end. Subsequently, the window method was used to define the subdivision of the dataset into two groups:

**Training Set:**  6 August 2019 00:00:00–28 August 2019 23:00:00 (23 days; 552 observations);
**Testing Set:**  29 September 2019 00:00:00–30 September 2019 23:00:00 (2 days; 48 observations).

As usual, the training set was used to build the data model while the testing set was used to evaluate the model performance.

To evaluate the predictive capabilities of the forecast model with respect to the testing set, the following metrics were used:

**Root mean square error**  computed as
  (1) $$RMSE=\sqrt{\frac{1}{N}\sum_{t=1}^{N}{\left({\hat{y}}_{t}-y_{t}\right)}^{2}}$$
**Mean absolute error**  computed as
  (2) $$MAE=\frac{1}{N}\sum_{t=1}^{N}\left|{\hat{y}}_{t}-y_{t}\right|$$
**Mean absolute percentage error**  computed as
  (3) $$MAPE=\frac{100}{N}\sum_{t=1}^{N}\frac{|{\hat{y}}_{t}-y_{t}|}{|y_{t}|}$$

where *N* is the number of forecasts, ${\hat{y}}_{t}$ is the forecast at time *t* and $y_{t}$ is the value of the testing set at time *t*.

### 2.6. Formalization of Statistical Methods

The available data for this study were a collection of observations related to well-defined soil, irrigation and atmospheric features, which were obtained through repeated measurements from specific sensors over time. These data were *time series*, i.e., they were sorted over time, sampled sequentially and presented a dependence between successive observations. Time series forecasting is usually a difficult problem to tackle; generally speaking, no model exists that can perform the best for all forecasting problems. However, statistical methods favor interpretability and have been demonstrated to be effective in many forecasting problems. So, we focused on stochastic models based on the Box and Jenkins procedure as starting models to tackle the peculiar forecasting task for the available data.

A *time series* is a finite set of observations of a certain phenomenon that evolves randomly, which are ordered in terms of time. Each observation $x_{t}$ can be seen as the realization of a random variable $X_{t}$. Therefore, a time series ${\left\{x_{t}\right\}}_{t\in T}$ is the realization of a family of random variables ${\left\{X_{t}\right\}}_{t\in T}$, i.e., a *stochastic process*.

Generally, time series are decomposed as follows:

(4) $$X_{t}=f\left(t\right)+\varepsilon_{t},$$

where the term *f* represents a deterministic law and ε represents the *stochastic part* of the series and obeys certain probability laws. In particular, more classical approaches denote *f* as the superposition of the trend, cyclical and seasonal components, i.e., f(t)=T(t)+C(t)+S(t), whereas in modern approaches, it is assumed that the deterministic component f(t) has already been eliminated via an estimation process and that $\varepsilon_{t}$ is a stochastic process with correlated components that depend on the past values of *x*, the past errors of ε itself and a further error component $w_{t}$:

$$\varepsilon_{t}=g\left(x_{t-1},x_{t-2},\ldots ,\varepsilon_{t-1},\varepsilon_{t-2},\ldots \right)+w_{t}$$

One such modern approach was developed by Box and Jenkins [30] at the beginning of the 1970s as a global approach for the statistical analysis of historical series, which aims to identify the probabilistic model to be used primarily for forecasting purposes, starting from what can be deduced from a given series. Without claiming to be exhaustive, we refer the interested reader to classical references [31]. The presence of exogenous variables means that time series cannot be trivially treated as non-stationary stochastic processes; nevertheless, non-stationary processes can be made stationary when they can be recast to polynomials of a certain degree, i.e., when the trend term in the time series can be approximated using an expression such as

(5) $$T_{t}=\beta_{0}+\beta_{1}t+\beta_{2}t^{2}+\ldots +\beta_{d}t^{d}.$$

## 3. Results

Now, we present the results of the forecasts obtained using different methods.

### 3.1. Decomposition Methods

One of the fundamental purposes of the classical analysis of time series is to decompose the series into its components, thereby isolating them in order to study them better. Furthermore, in order to apply a stochastic approach (e.g., AR, MA and ARIMA models) to time series, it is necessary to eliminate trends and seasonality in order to obtain a stationary process.

**The additive model with the moving average method**  The components of a time series are generally the trend, seasonality and residual. R allows us to determine and estimate these three components using different methods, including decomposed.

The decomposed method decomposes time series into their seasonality, trend and erratic components using the symmetric mean method and can be applied to both additive models and multiplicative models (which must be specified in the type = parameter c (“additive”, “multiplicative”)).

**Figure 4:**
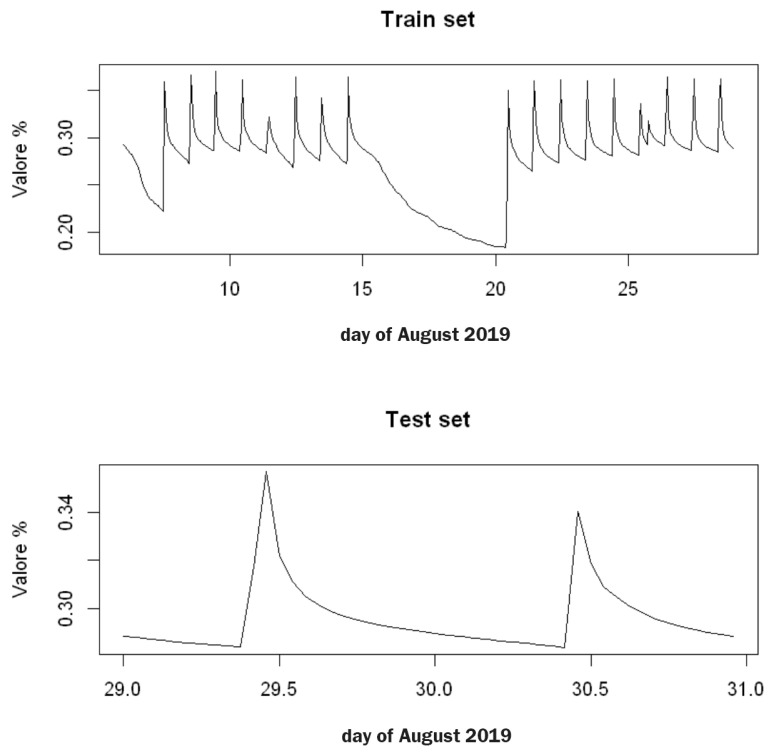
The time series data for soil water content in the A2 block at a depth of 50 cm. The data were split into a 23-day training set and a 2-day testing set. After data conversion obtained by the class ts, the following decomposition was obtained: > df.ts.suolo <- ts(df.suolo.agosto.ora[,2], start = c(6,1), end = c(30,24), frequency = 24)

It can be noticed in our data (for instance, see Figure 4), that the seasonal effects due to daily irrigation remained almost unchanged over the days. An additive model was considered in the initial phase of building an appropriate predictive model. In particular, this model allowed us to rewrite the $x_{t}$ series as follows:

(6) $$x_{t}=m_{t}+s_{t}+\epsilon_{t}.$$

**Figure 5:**
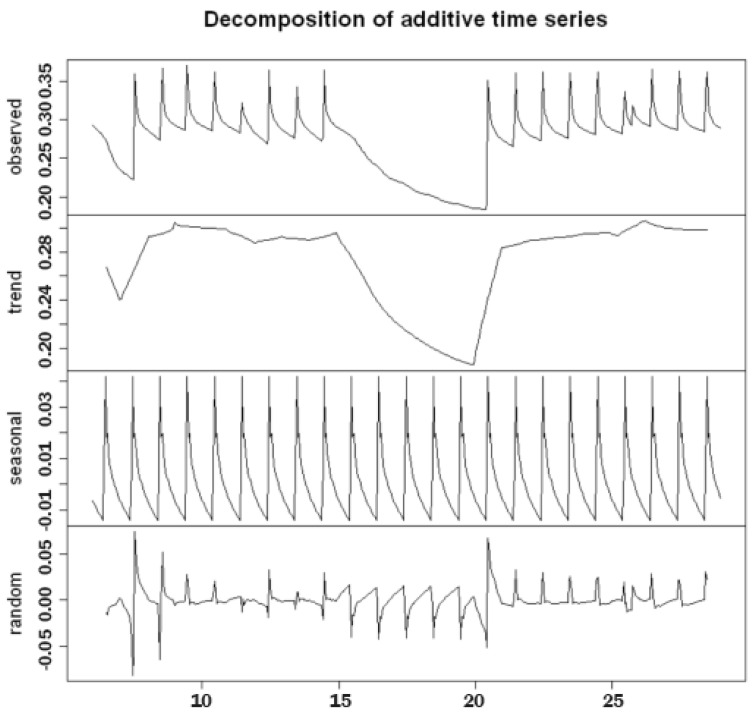
The additive decomposition by moving average method of time series data for soil water content in the A2 block at a depth of 50 cm in August 2019, obtained by the following code: > dec <- decompose(train, type = ’additive’)

**Figure 6:**
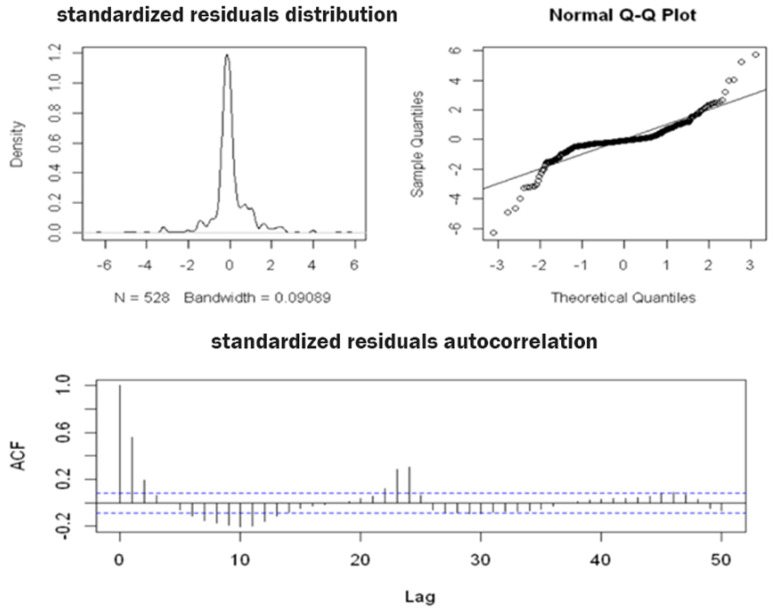
The diagnostic plots for the standardized residual components derived using the additive model with the moving average method for the soil water content time series data from the A2 block at a depth of 50 cm.

Figure 5 shows the results of the additive decomposition, highlighting the following:

- Excluding the seasonal and residual components, the **trend** component appeared to be almost stationary on days when irrigation took place and decreased on days when irrigation did not take place. Thus, in order to obtain a constant average, the series needed to be differentiated;
- The **seasonal component**, due to irrigation, was scanned at intervals of 24 h (daily) and had a constant variance over time, which confirmed that we needed an additive rather than a multiplicative model;
- The **residual component**, which should exhibit a similar behavior to white noise, instead exhibited a heteroscedastic behavior, with a variance that was not constant over time and peaked about every 24 h. Further confirmation can be obtained by looking at the plot of the distribution diagram and the Q–Q plot of the standardized residual components in Figure 6. Furthermore, the presence of correlation between the residual components is evident in the correlogram in the same figure, in which numerous significant lags can be observed. A verification of this was obtained using the Ljung–Box test: when the null hypothesis was true (i.e., the absence of autocorrelation), the LB statistic behaved asymptotically as a variable random chi-square statistic with k degrees of freedom, where k represents the number of considered lags.

From these considerations, it is possible to state that a simple additive model, such as that described by Equation (6), would not be suitable for the analysis of the available data.

**Holt–Winters exponential smoothing**  Exponential smoothing using the Holt–Winters method can be considered as a more sophisticated version of simple additive decomposition using the symmetric (or moving) average method, as seen in the previous section. The idea is to transform a moving average into an exponential average with one or more smoothing parameters. The R stats package provides the HoltWinters() function for the implementation of this method. Using this function with the added seasonality of 24-h periods, we obtained the following table:

**Holt–Winters Method**

**Soil Water Content at a Depth of 50 cm**

alpha
0.480902
beta
0
gamma
0.698756SSE on the training set0.088168

By calculating the statistic using R’s Box.Test() procedure and considering 24-h delays, a *p*-value $<2.2\times 10^{-16}$. was obtained, which confirmed the alternative hypothesis and, therefore, the presence of autocorrelation among the residual components. It followed that the residual components still contained useful information that was not included in the additive model.

**Figure 7:**
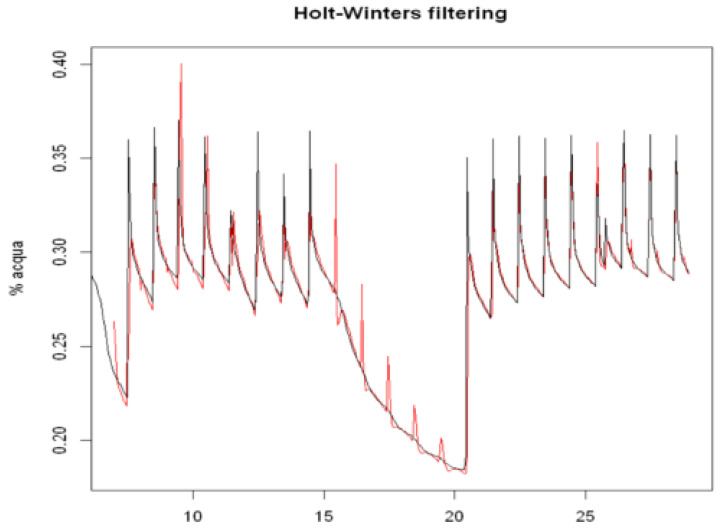
The trends in the time series data for soil water content in August 2019 (black) with respect to the time series obtained using the Holt–Winters exponential smoothing method on the training set (red line), which was performed in R using the following code: > model.train.hw <- HoltWinters(train)
> plot(model.train.hw).

The parameters α, β and γ were obtained by minimizing the sum of the squared residual components. They estimated the level variations, trend variations and the seasonal effects of the 24-h amplitude, respectively. Figure 7 illustrates the approximation of the time series obtained by the model on the training set. A null value of β indicated that the model did not detect any trends in the series. The sum of the squares of the residual components was 0.08817. To predict the soil water content for the 48 h following the last observation, we used the forecast() method, specifying the length of the forecast period (i.e., 48 h).

**Figure 8:**
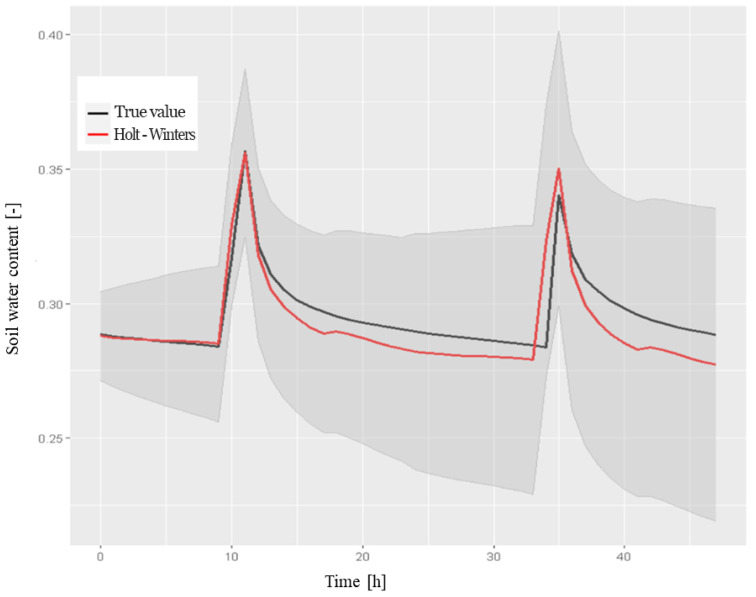
The 48-h forecast for soil water content in the A2 block at a depth of 50 cm obtained using the Holt–Winters method on the testing set with a confidence interval of 80%, which was obtained in R using the following code: > result.hw <- forecast(model.train.hw,48).

**Figure 9:**
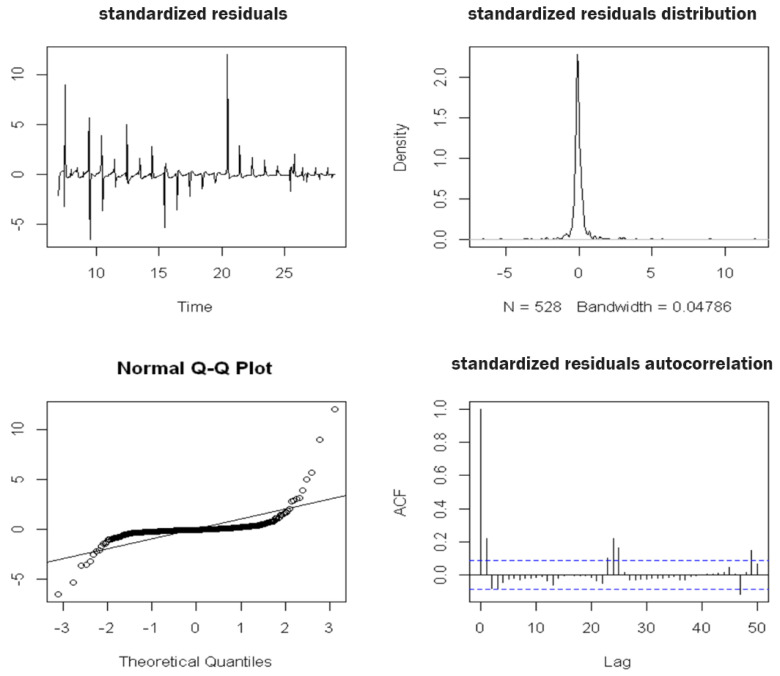
The diagnostic plots for the standardized residual components derived using the Holt–Winters exponential smoothing method for the soil water content time series data from the A2 block at a depth of 50 cm.

Figure 8 shows the results of the prediction model on the testing set, with a confidence interval of 80%. The forecast performance in terms of the previously defined metrics, compared to the average values from the testing set, was as follows: whereas Figure 9 reports the behavior of the diagnostic plots of the model.
**Performance Holt-Winters Method****Soil Water Content at 50 cm, 48 h Forecast**media test0.295670RMSE0.009268MAE0.007000MAPE2.371098

Even in this case, the residual components did not show white noise behavior. Notice the presence of numerous outliers, in particular on days 6 and 21, which were due to fact that the model was unable to emulate the peaks of the water content values when there was no irrigation on the previous day. To confirm this, Figure 8 shows that on those days, the peaks of the model (indicated in red) were much lower compared to the real values (denoted by the black line) and were still present on days when irrigation did not take place. Furthermore, there are numerous significant lags in the related correlogram, as shown in Figure 9. The Ljung–Box test confirmed the alternative hypothesis of the presence of correlation with a low *p*-value equal to $6.59\times 10^{-7}$.

### 3.2. Stochastic Methods

**SARIMA model**  This is a seasonal autoregressive moving average model used to better describe the generating function of time series. This model allows us to predict observed values with a reduced number of input variables, following the principle of parameter parsimony. It also provides the ability to extend forecasts over long time horizons. In order to identify the model, we followed the Box–Jenkins procedure.

1. *Stationarity of time series*. The first step was to check the stationarity of time series by analyzing correlograms and performing some statistical tests. When a time series was not stationary, it needed to be transformed into a stationary time series. The graph of the autocorrelation function for SWC suggested the presence of a linear trend and had high correlations among seasonal delays since a decreasing trend occurred during drying periods and peaks occurred after irrigation events: peaks repeated at intervals of about a regular amplitude equal to s = 24 and its multiples, which are called lag or seasonal delays, indicated the presence of the seasonal component of the series and contributed to the time series being non-stationarity on average. To make the time series stationary on average, we applied appropriate transformations. In particular, to remove the linear trend, the regular difference operator $\nabla^{d}$ of order d=1 was applied to the series.
2. *Identifying the SARIMA model* The next step was to analyze the graphs of the global and partial autocorrelation functions of the series, as shown in Figure 10, in order to identify significant delays in identifying hyperparameters. We noticed that the first order differentiation performed on the series eliminated the problem of the series being non-stationarity on average. From an analysis of the graph of the global autocorrelation function, we observed that the second delay was significantly different from the zero peak with regard to the regular part and was in correspondence with the second seasonal delay with regard to the seasonal part. Therefore, we could assume that the model that generated the series under analysis had a regular moving average component *q* between 0 and 2 and a seasonal moving average component *Q* between 0 and 2. The graph of the partial autocorrelation function shows a trend similar to that of the global autocorrelation function. Therefore, the series generator model had a regular autoregressive component *p* and seasonal *P* between 0 and 2. Using the auto.arima() method, we performed a grid search on the possible hyperparameters identified by the model and selected the best results based on statistic criteria, such as AIC and BIC. The optimal stochastic model for this series, as identified by this method, was a SARIMA process $\left(0,1,2\right){\left(2,0,1\right)}_{24}$.

**Figure 10:**
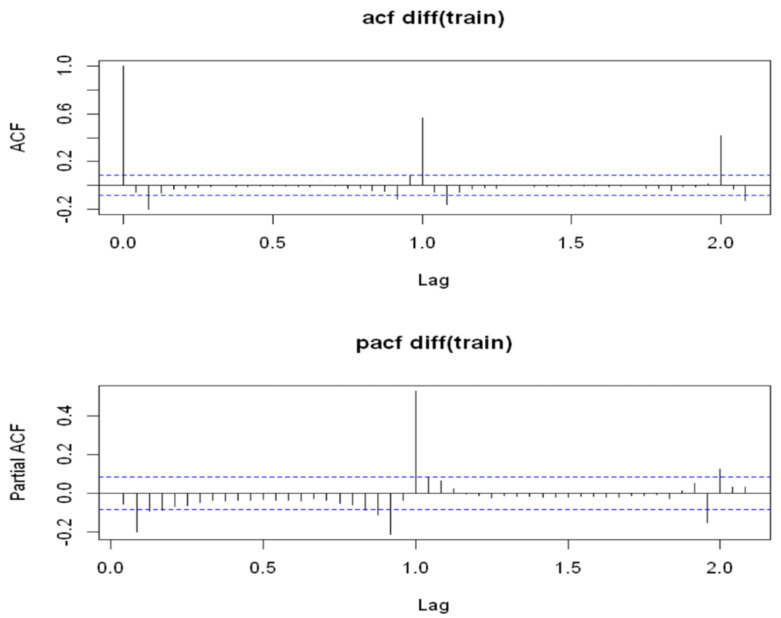
The correlograms of the autocorrelation function and partial autocorrelation function of the time series data for SWC in the A2 block at a depth of 50 cm following differentiation, which were obtained through the following functions of R: > train.diff = diff(train)> par(mfrow = c(2,1))> acf(train.diff,50, main = ’acf diff(train)’)> pacf(train.diff,50, main = ’pacf diff(train)’)An augmented Dickey–Fuller test confirmed the alternative stationarity hypothesis:> adf.test(train.diff, k = 24)Dickey-Fuller = −4.0635, Lag order = 26, *p*-value = 0.01.
3. *Parameter estimation* After identifying the possible hyperparameters of the series, we proceeded with the estimation of the model parameters. By applying a procedure based on the iterative method of maximum likelihood (command Arima()), a model was estimated using the parameters reported in the following table:
**SARIMA(0, 1, 2, 2, 0, 1, 24)****Water Content at a Depth of 50 cm****Parameters**CoefficientEstimations.e.*t*-Value*p*-Valuema1−0.19790.0416−4.76120ma2−0.21730.0423−5.13170sar11.29630.062320.81900sar2−0.30740.0568−5.41510sma1−0.89900.0477−18.83360**Statistics**Estimated $\sigma^{2}$0.000132Log Likelihood1670.18AIC−3328.36AICc−3328.21BIC−3302.49**Performance on the Training Set**RMSE0.011426MAE0.003525MAPE1.169268
4. *Model verification* After estimating a specific model, we checked its goodness and adequacy with respect to the observed data. We analyzed whether the parameter estimates were statistically significant, whether the residual components reflected the assumptions of the stochastic models and whether the *parsimony principle* was verified by the model, i.e., whether the number of parameters used was as low as possible. The first step was to estimate the coefficients of the model through the verification of the null hypothesis, i.e., H0:coeff=0. The sarima() method provides the Student’s t-test and the respective *p*-value for each parameter. Null *p*-values in the outputs, which are reported in the table, led to the rejection of the null hypothesis for all parameters. A further check to be carried out concerned an analysis of the residual components of the estimated SARIMA model. Firstly, we analyzed the residual plots and correlograms to evaluate their stationary behavior and determine whether they were unrelated, respectively. These diagnostic plots were produced by the same SARIMA procedure and are presented in Figure 11. We observed that the residual time series plot fluctuated around a constant value equal to zero, resulting in the series being stationary on average. The model residual components showed numerous peaks when irrigation occurred; however, these results were slightly lower compared to those obtained by the decomposition models. The plot of the autocorrelation function shows that few delays were significantly correlated, while the Q–Q plot shows a non-normal distribution of residual components due to the presence of outliers and diverging tails at the extremes. Finally, the last graph represents the value of the *p*-value associated with the Ljung–Box test statistic over time *k*, which always has a higher level of significance α=0.05. Consequently, the null hypothesis of non-correlation could be considered to be true.

**Figure 11:**
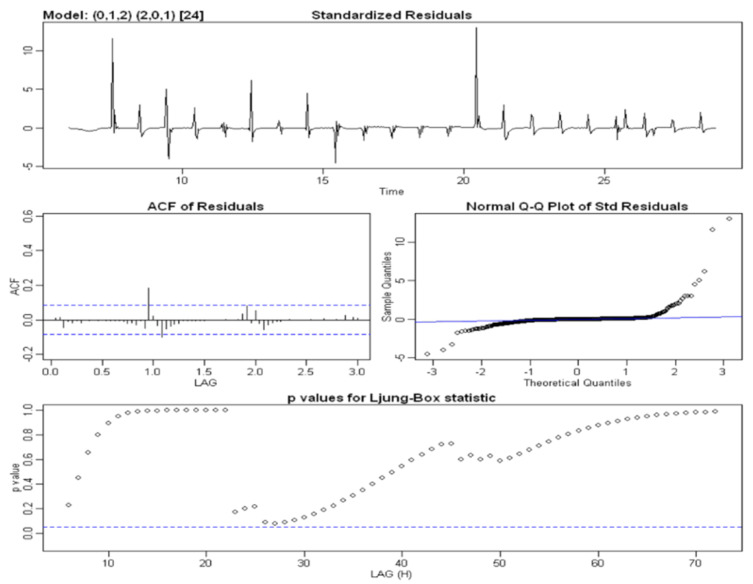
The diagnostic plots of the standardized residue components of the SARIMA model of the time series data for SWC in the A2 block at a depth of 50 cm, which were obtained using the following procedure in R: > model1 <- sarima(train,0,1,2,2,0,1,24, no.constant = TRUE).
5. *Evaluation of the predictive capabilities of the model* After verifying the model suitability, we tested its predictive capabilities by comparing the results obtained on the testing set and calculating the appropriate metrics. A 48-hour forecast was calculated using the forecast procedure.
  Figure 12 reports the results of the forecast with the respective 80% confidence interval and the comparison with the testing set. The metric values are shown in Table 1.

**Table 1:** The performance metrics for the SARIMA model (0,1,2,2,0,1,24) for SWC at a depth of 50 cm (48-h forecast).

Mean Test	0.295670
RMSE	0.004557
MAE	0.002045
MAPE	0.675001

**SARIMAX model**  The previous tests and graphs highlighted some drawbacks of the SARIMA model, as follows:

1. As shown in Figure 12, the model was not able to predict the exact time at which irrigation was performed and the relative SWC peak, assuming that it always occurred at 10:00 am. As previously observed in the testing set, irrigation was also performed at later times (generally 11:00 or 12:00). It was then considered necessary to add this information to the model using the dataset of the volume of water ($m^{3}$) used for irrigation.

**Figure 12:**
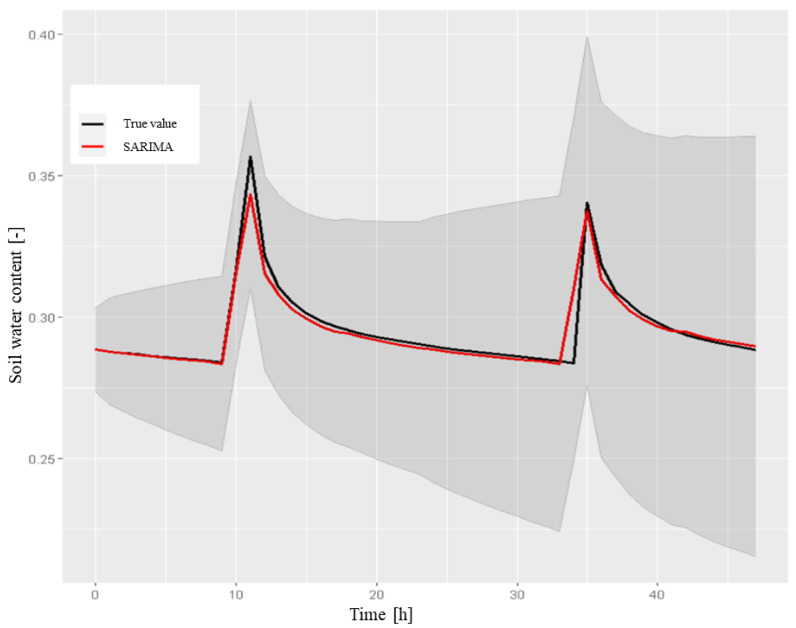
The 48-h forecast of SWC in the A2 block at a depth of 50 cm, which was obtained by the SARIMA model on the testing set, with a confidence interval of 80%. We used the following instructions: > sarima.forecast <- forecast(model1, h = 48).
2. The model did not consider any influences from external factors. As shown graphically, on 25 August, the rain had an impact on the SWC. This required a study of the significance of the atmospheric variables for the model.

We observed that the residual time series plot fluctuated around a constant value equal to zero. By adding external influencing factors to the SARIMA model, the time series and its forecast could be obtained by combining the model with a regression of these variables. In this way, we obtained seasonal autoregressive models that were integrated with the moving average with the addition of exogenous variables or SARIMAX. It was possible to specify the exogenous variables to be combined with the model using the xreg command in the Arima() procedure. However, during the forecast, it was necessary to provide the future values of the regressor parameters (i.e., the amount of water for irrigation and the weather forecast for the following days need to be known in advance). We inserted the irrigation and atmospheric data as exogenous variables after having them appropriately standardized on the testing set. The auto.arima function identified a process as an optimal SARIMA model $\left(1;1;1\right){\left(2;0;1\right)}_{24}$. To evaluate which parameters were relevant to the model, the Student’s t-test was performed using the arima() method. The results are shown in Table 2.

The classical parameters of the model were all significant, as well as the exogenous variables of irrigation and volume of rain. Despite the low *p*-value of the variable *atmospheric pressure*, there was no physical evidence that this variable should be included in the model. By estimating the SARIMAX model with the addition of significant regressor parameters, the following estimates were obtained, as shown in Table 3.

**Figure 13:**
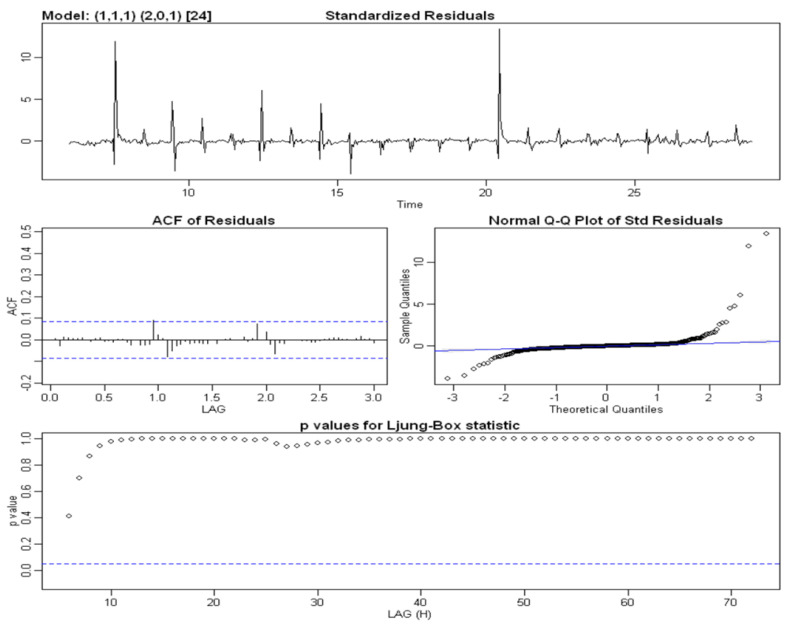
The diagnostic plots of the standardized residual components of the SARIMAX model of the time series data for SWC in the A2 block at a depth of 50 cm with exogenous rain and irrigation regressors, which were obtained through the following tools in R: > xreg <- cbind(irrigazione, meteo[,2])

Figure 13 shows the residual diagnostic plots. Here, even the residual component of the time series fluctuated around the null value and had corresponding peaks in irrigation schedules (in any case, this model contained than the previous model). The autocorrelation plot does not show any significantly correlated delays, unlike the simple SARIMA model. In addition, the *p*-value associated with the Ljung–Box test statistic appeared to be definitely above the significance level of 5% in contrast to that of the residual components of the previous model (Figure 11), which confirmed the non-correlation hypothesis.

**Figure 14:**
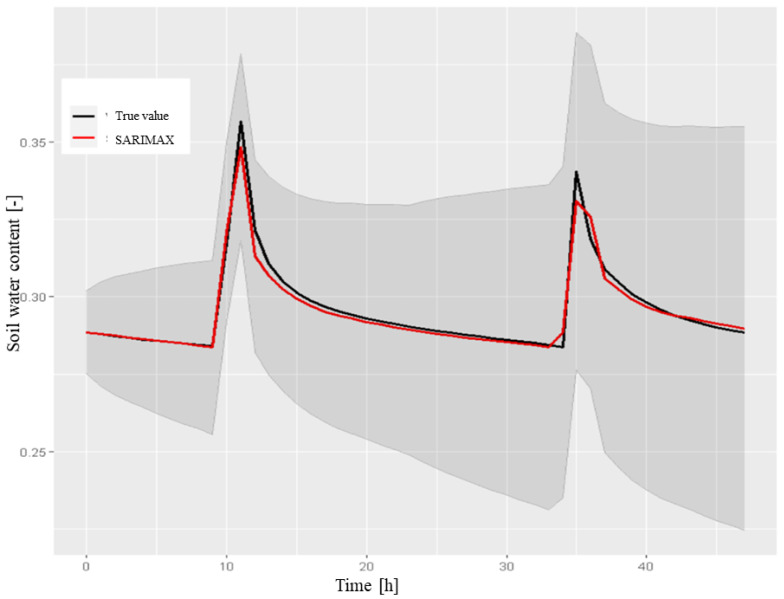
The 48-h forecast for SWC in the A2 block at a depth of 50 cm, obtained by the SARIMAX model with exogenous rain and irrigation regressors, compared to the testing set with a confidence interval of 80%, which was obtained by the following R instructions: > sarimax.forecast <- forecast(model2, xreg = xreg.test).

Finally, a 48-hour forecast was produced, which was obtained by providing the parameters as input future regressors. Figure 14 shows the results. Unlike the previous case, this model was able to predict the exact timings of SWC peak occurrence.

The computed metrics (reported in Table 4) confirmed the obtained improvement.

## 4. Discussions and Future Work

This paper illustrated how to use some well-established statistical methods to provide an accurate forecast for soil moisture content, temperature and electrical conductivity at two different depths, using standard TDR probes. A similar approach was used in [32] to schedule irrigation services using an LSTM neural network applied to the ERA5 climate reanalysis dataset. In an analogous framework, hydraulic parameters were estimated in [33] using neutron thermalization data.

The same dataset was adopted to obtain different forecasting models, which took advantage of some of the data properties and demonstrated the different model performances. To find which of the well-established statistical models produced the better forecasts from the available data, a comparison between the simpler and more complex models was performed. The Holt–Winters model could handle time series variability, trends and seasonality by smoothing out white noise weighting the most recent historic data. For this model, the tuning of only three parameters was required. The SARIMA models were more flexible because of their ability to adapt well to different time series patterns through the choice of seasonal and non-seasonal hyperparameters. They could perform better with the introduction of external regressor information by defining a SARIMAX model. However, the search for the optimal hyperparameters took more computational time compared to the simpler Holt–Winters method. In the described setting, where differences in irrigation schedules and atmospheric variables could impact the seasonality patterns of the time series, the SARIMAX model turned out to be the most appropriate and accurate in terms of the selected performance metrics.

Concerning the analysis of our results, although there was a certain daily periodicity in the analyzed dataset regarding soil water content (the trend of which was almost repetitive), days when soil irrigation was not carried out turned out to represent exceptions in the observed patterns. In these cases, there was a decreasing trend in the variable, for which the repetitiveness of the peaks failed. This occurrence was observable from the 15th to the 19th of August, as shown in Figure 4. Because daily seasonal models provide forecasts based on the previous values of the variable, when an irrigation day followed a non-irrigation day, the model could likely predict a peak where none were expected. Although adding a null regressor parameter could limit this peak, it was not enough to eliminate it.

To overcome this problem, autoregressive machine learning methods could be applied (both linear and nonlinear). This represents future work that will be carried out with the same data. Moreover, investigating the possibility of integrating these data-driven models with physics-based models, possibly in data assimilation or Bayesian frameworks (for instance, see [34,35,36,37]), is a promising field of research. Other techniques (for instance, see [38]) for optimizing hyperparameters in hybrid methods could also be investigated. Even more challenging is the idea of combining these techniques with more complex nonlocal models, which is now emerging in unsaturated flow modeling (e.g., [39]), coupled with sophisticated numerical techniques for nonlocal problems (as in [40,41]).

## 5. Conclusions

The paper illustrated how to apply some well-established statistical techniques (ARIMA) and seasonal extensions (SARIMA) to construct data-driven models for forecasting soil water content and salinity in the particular context of irrigation with reclaimed water. After a proper pre-processing phase and an exploratory analysis of the collected time series data (including soil water content and salinity data at a depth of 50 cm beneath the soil surface, atmospheric data and daily irrigation data), different approaches were considered to include data characteristics, such as seasonality and additional climate data. The obtained forecasting models showed good accuracy and a good interpretability of the results and represented a novelty in the context of irrigation with reused water from agro-industrial origins, particularly in terms of properly planning water resources according to climate changes. 

## Figures and Tables

> class(df.ts.suolo)

’ts’

> train <- window(df.ts.suolo, start = c(6,1), end = c(28,24))

> test <- window(df.ts.suolo, start = c(29,1), end = c(30,24))

> length(train)

552

> length(test)
48.

> plot(dec)
This shows the behavior of the time series and its trend, seasonal and random components.

> xreg.ts <- ts(xreg, start = c(6,1), frequency = 24)

> xreg.train <- window(xreg, start = c(6,1), end = c(28,24))

> xreg.test <- window(xreg, start = c(29,1), end = c(30,24))

> model2 <- sarima(train,1,1,1,2,0,1,24, xreg = xreg.train, no.constant = TRUE).

**Table 2:** The performance of the SARIMAX model (1,1,1,2,0,1,24) for SWC at a depth of 50 cm.

Parameters				
Coefficient	Estimate	s.e.	*t*-Value	*p*-Value
ar1	0.3933	0.1275	3.0843	0.0021
ma1	−0.6386	0.1043	−6.1219	0.0000
sar1	1.2698	0.0655	19.3838	0.0000
sar2	−0.2767	0.0554	−4.9995	0.0000
sma1	−0.9355	0.0764	−12.2456	0.0000
irrigazione	0.0071	0.0007	10.8599	0.0000
meteo.pioggia	0.0050	0.0022	2.2613	0.0241
meteo.temperatura	0.0008	0.0007	1.0528	0.2929
meteo.umidita	0.0003	0.0002	1.7974	0.0728
meteo.radiazione	0.0034	0.0027	1.2462	0.2132
meteo.vento.velocita	−0.0004	0.0016	−0.2547	0.7991
meteo.vento.raffica	0.0004	0.0008	0.4811	0.6306
meteo.vento.direzione	0.0000	0.0000	−1.5981	0.1106
meteo.bagnatura.fogliare.sup	0.0001	0.0001	1.2025	0.2297
meteo.bagnatura.fogliare.inf	−0.0002	0.0001	−1.5021	0.1337
meteo.pressione.atmosferica	0.0036	0.0001	24.8314	0.0000
meteo.punto.di.rugiada	−0.0006	0.0007	−0.9643	0.3353
meteo.Etp	−0.0191	0.0130	−1.4719	0.1416

**Table 3:** The performance of the SARIMAX model (0,1,2,2,0,1,24) for SWC at a depth of 50 cm.

Parameters				
Coefficient	Estimate	s.e.	*t*-Value	*p*-Value
ar1	0.4934	0.1143	4.3151	0
ma1	−0.6923	0.0943	−7.3425	0
sar1	1.2766	0.0559	22.8501	0
sar2	−0.2808	0.0511	−5.4956	0
sma1	−0.9471	0.0578	−16.3963	0
irrigazione	0.0089	0.0006	14.9233	0
meteo.pioggia	0.0045	0.0018	2.5251	0.0118
**Statistics**				
Estimate $\sigma^{2}$		0.000094		
Log Likelihood		1762.72		
AIC		−3509.44		
AICc		−3509.17		
BIC		−3474.94		
**Performance on the Training Set**				
RMSE		0.009627		
MAE		0.002935		
MAPE		0.989883		

**Table 4:** The performance of the SARIMAX model (1,1,1,2,0,1,24) for SWC at a depth of 50 cm (48-h forecast).

Mean Test	0.295670
RMSE	0.003042
MAE	0.001951
MAPE	0.631653

## Data Availability

Data are available upon request after emailing the corresponding author and/or G.A. Vivaldi.
